# Erie County Medical Society

**Published:** 1867-03

**Authors:** Thos. M. Johnson

**Affiliations:** Sec’y.


### Erie County Medical Society.

The regular Annual Meeting of Erie County Medical Society was held on the 8th day of January, 1867, at the rooms of the Buffalo Medical Association in this city. The usual business of these meetings was transacted, and the following officers elected for the ensuing year:

President, Dr. J. R. Lothrop; Vice President, Dr. John Boardman; Secretary, Dr. Thomas M. Johnson; Treasurer, Dr. Wm. Ring; Librarian, Dr. James B. Samo.

Primary Board—Drs. H. S. Taft, W. C. Phelps and Frank Abbott.

Censors—Drs. S. W. Wetmore, S. F. Mixer, Thomas Lothrop, P. H. Strong, John Hauenstein.

Dr. George Abbott, of White's Corners, informed the meeting that by a recent apportionment Erie county is entitled to five members in the Assembly, and that therefore this Society is entitled to another delegate to the State Medical Society. An election was held when Dr. George Abbott was elected such delegate.

The following gentlemen were admitted members of the Society:—Dr. M. E. Shaw of Buffalo, Dr. Lapp of Clarence.

Dr. George Abbott read an elaborate paper upon Diphtheria, for which he received a vote of thanks from the Society, and a request of a copy for publication.

Thos. M. Johnson, Sec'y.

